# Factors associated with the use of home-visit nursing services covered by the long-term care insurance in rural Japan: a cross-sectional study

**DOI:** 10.1186/1471-2318-13-1

**Published:** 2013-01-02

**Authors:** Masayo Kashiwagi, Nanako Tamiya, Mikiya Sato, Eiji Yano

**Affiliations:** 1 Department of Health Services Research, faculty of medicine, University of Tsukuba, 1-1-1 Tenno-dai, Tsukuba Ibaraki, 305-8575, Japan; 2 Department of Hygiene and Public Health, Teikyo University, School of Medicine, 2-11-1 Kaga, Itabashi-ku, Tokyo, 174-8605, Japan

**Keywords:** Home-visit nursing service, Long-term care insurance, Care management, Community-based service, Care-needs level, Living alone, Income level, Home-help service

## Abstract

**Background:**

In Japan, there is a large increase in the number of elderly persons who potentially need home-visit nursing services (VNS). However, the number of persons using the VNS has increased only little in comparison to the number of individuals who use home social services, which are also covered by the Long-Term Care Insurance (LTCI) system. This cross-sectional study investigated the predictors of the VNS used under the LTCI system in Japan.

**Methods:**

We used 1,580 claim data from all the users of community-based services and 1,574 interview survey data collected in 2001 from the six municipal bodies in Japan. After we merged the two datasets, 1,276 users of community-based services under the LTCI were analyzed. Multiple logistic regression models stratified by care needs levels were used for analysis.

**Results:**

Only 8.3% of the study subjects were VNS users. Even among study participants within the higher care-needs level, only 22.0% were VNS users. In the lower care level group, people with a higher care level (OR: 3.50, 95% CI: 1.50–8.93), those whose condition needed long term care due to respiratory or heart disease (OR: 4.31, 95% CI: 1.88–89.20), those whose period of needing care was two years or more (OR: 2.01, 95% CI: 1.14–3.48), those whose service plan was created by a medical care management agency (OR: 2.39, 95% CI: 1.31–4.33), those living with family (OR: 1.86, 95% CI: 1.00–3.42), and those who use home-help services (OR: 2.12, 95% CI: 1.17–3.83) were more likely to use the VNS. In the higher care level group, individuals with higher care level (OR: 3.63, 95% CI: 1.56–8.66), those with higher income (OR: 3.79, 95% CI: 1.01–14.25), and those who had regular hospital visits before entering the LTCI (OR: 2.36, 95% CI: 1.11–5.38) were more likely to use the VNS.

**Conclusions:**

Our results suggested that VNS use is limited due to management by non-medical care management agencies, due to no caregivers being around or a low income household. The findings of this study provide valuable insight for LTCI policy makers: the present provision of VNS should be reconsidered.

## Background

As the number of older persons in need of long-term care services increase, community based services continue to hold the be the predominant position among elderly care services in developed countries. Japan is the most rapidly aging country in the world. In 2010, the proportion of those aged 65 and over in the total population was 22.7% [1]. It is estimated that it will reach 40.5% by 2055, with 1 in 2.5 people being 65 years old and over [1]. Because of rising medical expenses caused by these dramatic changes in demographics, the Japanese government developed a policy to substitute long hospital stay with a community-based service. In 2000, a long term care insurance (LTCI) that aimed at providing long term care for the elderly population by integrating health services and social services was introduced in Japan [2,3].

Under the LTCI, persons certified by the municipal government as in need of care or support will receive benefits in the form of the following services. Long-term care benefits consist of mainly two services: community-based services and facility services. The two main types of community-based services are (1) health services: home-visit nursing services (VNS), home-visit rehabilitation services, and management guidance for in-home care and (2) social services: home-help services, home-bath services, and rental service for assistive devices. These services have a fixed fee determined by the central government (see Table 1). Each insured individual pays 10% of the eligible charges for services as a co-payment. In order to receive services from the LTCI insured person need to obtain and apply for certification of needed long-term care. The degree for long-term care requirement is classified into six care-needs levels. The maximum payment is fixed by each level. With the increasing number of elderly persons and because of the health policy, the home care needs of the frail elderly persons are increasing in Japan. Home health services by a physician are covered exclusively by the health insurance instead of by the LTCI. However, the VNS, one of the home health services, is covered mainly by the LTCI. Except for some cases (terminally ill patients with cancer, patients with incurable disease, acute exacerbation of disabling conditions, or individuals under 40), the VNS users are covered by the LTCI and not by health insurance. The Japanese Health, Labour, and Welfare Ministry reported that approximately 80% of the VNS users were insured by the LTCI in 2008.

**Table 1:** Community-based services and fees for each service in April 2000 (Unit: Japanese Yen)

**Community-based service**		**Fixed fee**
Visiting nurse service*	from visiting nurses agency	8,300
	from hospital	5,500
Home-help service*	Caregiving	4,020
	Housekeeping	2,220
Home bath service		12,500
Home-visit rehabilitation		5,500

* For visits 30 to 60 minutes long.

The number of persons using the VNS under the LTCI was 458,300 in the FY2010. That is only 10.9% of the total community-based services users covered by the LTCI. The total number of the community-based services users has increased about 1.9-fold since the FY2001. However, the number of persons using the VNS increased at a lower rate (1.3-fold) compared to the social services, such as home-help services (1.8-fold) and day care services (2.2-fold) [4]. The difference between the VNS and social services is that the VNS provides health services under medical doctor’s guidance. Pursuant to progressive population aging, national health services expenditures have been increasing [5]. The number of outpatients also has been increasing with age [6]. However, the number of the VNS users has hardly changed since the introduction of the LTCI system. We are concerned that the use of the VNS covered by the LTCI is limited due to systemic reasons. We hypothesize that the reasons for the lower use of the VNS under the LTCI include the following:

First, the care management system of the LTCI might contribute to the low use of the VNS. In order to use the services under the LTCI, the insured person needs to prepare a long-term care service plan (care plan), which is a combination of several types of services, depending on one’s need for care or support. The care plan is established by the care managers of care management agencies. After the care plan is created, the care management agency contracts with service providers on behalf of the insured person. A study showed that the care plans prepared by care managers of medical corporations mostly consist of a combination of health services and social services, but the great majority of care plans prepared by care managers of non-medical corporations utilize a combination of social services only [7]. These reports suggest that the use of the VNS may be related to the corporation type of the care management agency. However, so far, there is no further research examining this relation.

Second, the VNS fees are highest for community-based services covered by the LTCI (see Table 1). Service users are requested to pay 10% co-payment, except for the few who receive public livelihood assistance. For the low income elderly population, this 10% co-payment is a heavy burden. As a result, clients and their families, regardless of their actual needs, may choose more affordable services such as home-help services that cost less than the VNS under the LTCI [8]. The relation between the VNS and income level has not been reported previously.

Ten years after the establishment of the LTCI, the Japanese government promotes the VNS in preparation for the super-aging society and the high rate of elderly mortality. The VNS is the centerpiece of the health insurance reform or the long-term care insurance system. Therefore, it is important to identify the factors related to the use of the VNS through empirical studies. However, there are only a few empirical studies investigating the factors associated with VNS use in Japan. One study reported that VNS users authorized to receive the VNS by care managers had higher rates of medical treatment and severe dementia levels compared to non-users [9]. Another study reported that LTCI clients with higher care needs used the VNS more [10]. However, these previous studies did not investigate the influence of service provisions under the LTCI on the VNS use.

In other countries, many studies have found significant relations between elderly persons’ use of home health services, and their physical characteristics (e.g., functional status), household characteristics (e.g., income level), ethnicity, and geographic location [11-16]. Although these results were almost concordant, they included all the services and not only the VNS. Moreover, the LTCI make the community-based services provision quite unique in Japan; generally service use is arranged by a care-manager, and services use is restricted by the maximum payment. Therefore, we selected corporation type of care management agency, care-needs levels, and income as the main variables focusing on the Japanese LTCI system.

The aim of our study was to investigate the predictors of the VNS use covered by the Japanese LTCI using the claim data and surveys conducted on the insurers of the LCTI in six Japanese rural towns. We combined important enabling factors related to the LTCI system, such as the organization type of the care management agency, income level, care-needs level, and family caregiver’s situation [9-16], with predisposing factors in this study. Our results could contribute toward meeting the needs for the VNS use better.

## Methods

### Subjects

A survey was conducted in six rural towns in Kagoshima prefecture in the southeast of Japan. Overall, 22.6% of the population was 65 years and older in 2001, which was much higher than the national average (17.3%, 2001). We obtained electronic claims data on all users from the six municipal bodies for the period of November 1 to November 30, 2001. The claims data included all of the elderly who used any of the services covered by the LTCI (n = 2,158). Of these, 1,580 lived at their homes and used some form of community-based service. The utilization of the VNS was retrieved from the LTCI claim data as an outcome variable. We also obtained data on the utilization of home-help service, the user’s age as of November 1^st^, 2001, sex, care-needs levels (There are six care-needs levels, including the care-support level. Care-needs level 5 means that the patient has highly critical needs), and the corporation type of the care management agency (medical corporation or non-medical corporation). We identified those as predisposing factors in this study.

### Interview survey

To collect additional information about the characteristics of users and family caregivers, we used data from the interview surveys of the same set of participants from the municipal government. In this survey, public health nurses of the municipal government conducted an interview on the service users and their primary caregivers in November 2001. We utilized the interview survey data of 1,574 home care users (with a response rate of 99.7%). From these interviews, we incorporated the data on user’s sex, user’s age, household income levels (5 income levels, income level 1 referring to the lowest poverty income level), household composition (single, couple, or other), marital status, conditions that caused the need for long term care (single -choice among these category: respiratory disease, heart disease, incurable disease, stroke, fracture, rheumatism, dementia, or other), regular hospital visits before entry into the LTCI, period of needing care, the family caregiver’s sex, the family caregiver’s age, clients living or not living with family, the family caregiver’s kinship, and home-help service used.

### Statistical analysis

We merged the claims data (n = 1,580) and the interview survey data (n = 1,574). As a result, we obtained 1,530 community-based service user data profiles. We excluded missing income levels (n = 17) and household income level 1 (i.e., the lowest poverty level) (n = 60) from the analysis, because income level 1 users are not required to pay the 10% co-payment fee for the service used. Finally, 1,276 users of community-based services covered by the LTCI were analyzed. To define the factors of the VNS use, bivariate analyses were conducted by using the chi-squared test or the Wilcoxon rank sum test (Table 2). From Table 3, we can see that the two groups are in many aspects different (sex, age, regular hospital visits before entry into the LTCI, period of needing care, caregiver’s sex, caregiver/applicant dyad, clients living with family or not, corporate type of care management agency, and VNS use). Therefore, we did a separate analysis of each care-needs level group. Moreover, maximum payment increases more when the care-needs level reaches the level 3. Maximum payment is important as enabling factors for service use under the LTCI. In order to control the influence of care-needs level on the use of VNS, we first checked the distribution of the numbers of VNS users according to different care-need levels. We needed to do this because more specific factors related to service use might have been masked by the strong effect of the care-needs level. Then, we stratified the six care levels into lower care-needs level subgroups (care support level or care needs level 1-2) and higher care-needs level subgroups (care needs level 3 and 5). Kato [17] also used this categorizing strategy.

**Table 2:** Characteristics of study participants by use of VNS (N = 1,276)

	**All**		**VNS**				***χ***^**2**^	**p-value**	
			**Non-use**		**Use**				
	**N**	**%**	**n**	**%**	**n**	**%**			
**All**	1276	100	1170	91.7	106	8.3			
**Sex**									
Female	936	73.4	871	93.1	65	6.9	8.5645	0.0034**	
Male	340	26.7	299	87.9	41	12.1			
**Age**									
< 65	12	1.0	11	91.7	1	8.3		0.9729	†
65–74	159	13.5	144	90.6	15	9.4			
75–84	551	46.8	511	92.7	40	7.3			
85≦	456	38.7	413	90.6	43	9.4			
**Household Income Level**									
Lower income level	1210	95.9	1116	92.2	94	7.8	8.6624	0.0032**^1^	
Income level 2	856	67.8	791	92.4	65	7.6			
Income level 3	354	28.1	325	91.8	29	8.2			
Higher income level(paying personal tax)	52	4.1	42	80.8	10	19.2			
Income level 4	40	3.1	33	82.5	7	17.5			
Income level 5	12	1.0	9	75.0	3	25.0			
**Care-Needs Level**									
Lower care-needs level	1067	83.6	1007	94.4	60	5.6		< 0.0001**^2^	†
Support-requested Level	305	28.6	297	97.4	8	2.6			
Care-Needs Level 1	577	45.2	547	94.8	30	5.2			
Care-Needs Level 2	185	14.5	163	88.1	22	11.9			
Higher care-needs level	209	16.4	163	78.0	46	22.0			
Care-Needs Level 3	98	7.7	83	84.7	15	15.3			
Care-Needs Level 4	65	5.1	53	81.5	12	18.5			
Care-Needs Level 5	46	3.6	27	58.7	19	41.3			
**Conditions that caused a need for LTC**									
Respiratory or heart disease	72	5.7	59	81.9	13	19.1	9.8439	0.0017**^3^	
Heart disease	47	3.7	44	93.6	3	6.4			
Respiratory disease	25	2.0	15	60.0	10	40.0			
Others	1196	94.3	1105	92.4	91	7.6			
Stroke	318	25.1	283	89.0	35	11.0			
Fracture	73	5.8	72	98.6	1	1.4			
Rheumatism	300	23.7	285	95.0	15	5.0			
Incurable disease	38	3.0	34	89.5	4	10.5			
Aging	105	8.0	92	90.2	10	9.8			
Dementia	95	7.5	88	92.6	7	7.4			
Others	268	21.1	249	92.9	19	7.1			
Unclear	2	0.2	2	100.0	0	0.0			
**Regular hospital visits before entry to the LTCI**									
Yes	553	43.3	492	89.0	61	11.0	9.5041	0.0021**	
No	723	56.7	678	93.8	45	6.2			
**Period of needing care**									
< 2 years	546	43.4	515	94.3	31	5.7		0.0035*^4^	†
< 1 year	168	13.3	160	95.2	8	4.8			
1 year≦, < 2 years	378	30.1	355	93.9	23	6.1			
2 years≦	712	56.6	639	89.8	73	10.2			
2 years≦, < 3 years	146	11.6	133	91.1	13	8.9			
3 years≦, < 5 years	213	16.9	185	86.8	28	13.2			
5 years≦	353	28.1	321	90.9	32	9.1			
**Caregiver’s sex**									
Female	587	72.2	513	87.4	74	12.6	5.0630	0.0244*	
Male	226	27.8	210	92.9	16	7.1			
**Caregiver’s Age**									
< 65	30	3.7	25	83.3	5	16.7		0.7008	†
65–74	399	49.3	361	90.5	38	9.5			
75–84	211	26.1	181	85.8	30	14.2			
85≦	169	20.9	152	89.4	17	10.1			
**Caregiver/applicant dyad**									
Wife/Husband (living with)	304	24.5	266	87.5	38	12.5	10.1729	0.0014**^5^	
Others	935	75.6	872	93.3	63	6.7			
Son/Daughter (living with)	178	14.4	162	91.0	16	9.0			
Daughter/Son-in-law (living with)	85	6.9	76	89.4	9	10.6			
Other relatives living together	16	1.3	15	93.8	1	6.3			
Others	656	53.0	619	94.4	37	5.6			
**Clients living with family or not**									
Living alone	539	42.7	516	95.7	23	4.3	19.6464	< 0.0001**	
Living with family	723	57.3	642	88.8	81	11.2			
**Corporation type of care management agency**									
Medical	357	28.0	316	88.5	41	11.5	6.5698	0.0104*^6^	
Non-medical	919	72.0	854	92.9	65	7.1			
Social	773	60.6	721	93.3	52	6.7			
Others	146	11.4	133	91.1	13	8.9			
**Home-Help Service use**									
Yes	417	32.7	376	90.2	41	9.8	1.8910	0.1691	
No	859	67.3	794	92.4	65	7.6			

* p < 0.05 ** p < 0.01 † Wilcoxon rank sum test, other using the chi-squared test.

1 Lower income level versus Higher income level.

2 Lower care-needs level versus Higher care-needs level.

3 Respiratory or heart disease versus Others: classification utilizing the point of view of nursing care.

4 < 2 years versus 2 years≦.

5 Wife/Husband versus Others.

6 Medical versus Non-medical.

**Table 3:** Lower care level and higher care level by characteristics of study participants

	**Lower Care-needs level Group**^**1**^**(n = 1,067)**		**Higher Care-needs level Group**^**2**^**(n = 209)**		***χ***^**2**^	**p-value**
	**n**	**%**	**n**	**%**		
**Sex**						
Female	830	88.7	106	11.3	65.5268	< .0001**
Male	237	69.7	103	30.3		
**Age**						
< 75	143	69.8	62	30.2	34.2783	< .0001**
75≦	924	86.3	147	13.7		
**Income Level**						
Lower income level^3^	1020	84.3	190	15.7	3.1929	0.0740
Higher income level^4^	39	75.0	13	25.0		
**Conditions that caused a need for LTC**						
Respiratory or heart disease	60	83.3	12	16.7	0.0038	0.9505
Others	1000	83.6	196	16.4		
**Regular hospital visits before entry into the LTCI**						
Yes	429	77.6	124	22.4	26.0288	< .0001**
No	638	88.2	85	11.8		
**Period of needing care**						
< 2 years	481	88.1	65	11.9	14.0790	0.0002**
2 years≦	571	80.2	141	19.8		
**Caregiver’s sex**						
Female	420	71.6	167	28.4	15.7563	< .0001**
Male	192	85.0	34	15.0		
**Caregiver/applicant dyad**						
Wife/Husband (living with)	205	67.4	99	32.6	74.8750	< .0001**
Others	829	88.7	106	11.3		
**Clients living with family or not**						
Living alone	517	95.9	22	4.1	105.0940	< .0001**
Living with family	537	74.3	186	25.7		
**Corporation type of care management agency**						
Medical	282	79.0	75	21.0	7.7551	0.0054**
Non-medical	785	85.4	134	14.6		
**VNS use**						
Yes	60	56.6	46	43.4	61.6074	< .0001**
No	1007	86.1	163	13.9		
**Home-Help Service use**						
Yes	344	82.5	73	17.5	0.5741	0.4486
No	723	84.2	136	15.8		

* p < 0.05 ** p < 0.01 Using the chi-squared test.

1 Lower care-needs level group: Support-required/care levels 1–2.

2 Higher care-needs level group: Care levels 3–5.

3 Lower income level: not paying personal income tax.

4 Higher income level: paying personal income tax.

A multiple logistic regression analysis using stepwise variable selection method was used to examine the predictors of the VNS use for each care-needs level group, after checking for multicollinearity. The independent variables identified in bivariate analyses as predictors for the regression models are those with p-values less than 0.2. Relationship strengths were explained using crude odds ratio (OR) and 95% confidence interval (95% CI). The inclusion and exclusion criteria for the stepwise regression were both 20% in order to include additional variables in the final model, and thus allowing some protection against confounders. In the final model, p-values less than 0.05 were considered to be statistically significant.

The Hosmer-Lemeshow test [18] was used for the goodness-of-fit test in the model. The 95% CIs were based on the likelihood test statistics. All the analyses were performed with SAS software, version 9.13 [19].

### Ethical issues

The internal ethical review board of the University of Tsukuba, the university in which the first author was affiliated, approved this study. We performed the study within the terms of our contract with the local authorities of the six towns that we studied. The contract pertained to the secondary use of the public database.

## Results

### Demographic characteristics

The demographic characteristics of the study participants are shown in Table 2. Of the 1,276 users of community-based services covered by the LTCI, 106 (8.3%) used the VNS, 936 (73.4%) were female, 1,007 (85.5%) were 75 years of age and over, and 1,210 (95.9%) belonged to a lower income level group (i.e., individuals who do not have to pay personal taxes). Care-needs level 1 is the largest level, which accounts for 45.2%. The most frequent health reason for using LTCI was the presence of a stroke (25.1%), and 43.3% of study participants were regularly visiting a hospital before their entry into the LTCI. Of the study participants, 63.7% had a caregiver, 72.2% of these caregiver were female. The majority (57.3%) of the users were living their families. Three hundred fifty seven (28.0%) users had their care plan created by care management agencies belonged to a medical corporation. Four hundred and seventeen (32.7%) were home-help service users.

### Factors related to VNS use: results of bivariate analysis

The characteristics of the VNS user and non-user groups are shown in Table 2. As the care-needs level increased, the VNS was utilized more. The following differed significantly in the VNS user group: more male (12.1% vs 6.9%, *p* = 0.0034), higher income level (19.2% vs 7.8%, *p* = 0.0032), higher care-needs level (22.0% vs 5.6%, *p* < 0.0001), the condition that caused the need for long term care was a respiratory or heart disease (19.1% vs 7.6%, *p* = 0.0017), regular hospital visits before entry into the LTCI (11.0% vs 6.2%, *p* = 0.0021), period of needing care was two years or more (10.2% vs 5.7%, *p* = 0.0035), having one’s wife/husband as a caregiver (12.5% vs 6.7%, *p* = 0.0014), clients living with family (11.2% vs 4.3%, *p* < 0.0001), and medical care management agency created the care plan (11.5% vs 7.1%, *p* = 0.0104). The user’s age was not significantly related to the use of VNS.

Table 3 shows characteristics of the differences between lower care-needs level groups (care support or care needs levels 1 to 2) and higher care-needs level groups (care-needs levels 3 to 5) using the chi-squared test. As a result, all independent variables, except for income level, conditions that caused a need for LTC and home-help service use, were significantly associated with the care-needs level. We then stratified the six care-needs levels into lower and higher care-needs level groups and examined the association between the VNS and client characteristics. Through the chi-squared test or the Fisher’s exact test, we identified four factors in the lower care-needs level group and one factor in the higher care-needs level group as predictors associated with VNS use (Table 4). In the lower care-needs level group, the following factors were related to the likelihood of VNS use: the condition that caused the need for long term care was a respiratory or heart disease (*p* = 0.0011), period of needing care was two years or longer (*p* = 0.0158), client living with family (*p* = 0.0077), and medical care management agency created the care plan (*p* = 0.0314). In the higher care-needs level group, individuals with higher income (*p* = 0.0314) were related to the likelihood of VNS use.

**Table 4:** Factors related to VNS use after stratified care levels

	**Lower care-needs level group**							**Higher care-needs level group**						
	**VNS non-use (n = 1007)**		**VNS use (n = 60)**		***χ***^**2**^	**p-value**		**VNS non-use (n = 163)**		**VNS use (n = 46)**		***χ***^**2**^	**p-value**	
	**n**	**%**	**n**	**%**				**n**	**%**	**n**	**%**			
**Sex**														
Female	788	94.9	42	5.1	2.2318	0.1352		83	78.3	23	21.7	0.0122	0.9122	
Male	219	92.4	18	7.6				80	77.7	23	22.3			
**Age**														
< 75	134	93.7	9	6.3	0.1399	0.7084		52	83.9	10	16.1	1.7758	0.1827	
75≦	873	94.5	51	5.5				111	75.5	36	24.5			
**Income Level**														
Lower income level^1^	965	94.6	55	5.4		0.2698	†	151	79.5	39	20.5	4.6317	0.0314*	
Higher income level^2^	35	89.7	4	10.3				7	53.9	6	46.2			
**Conditions that caused a need for LTC**														
Respiratory or heart disease	50	83.3	10	16.7		0.0011**	†	9	75.0	3	25.0		0.7249	†
Others	951	95.1	49	4.9				154	78.6	42	21.4			
**Regular hospital visits before entry into the LTCI**														
Yes	400	93.2	29	6.8	1.7467	0.1863		92	74.2	32	25.8	2.5607	0.1096	
No	607	95.1	31	4.9				71	83.5	14	16.5			
**Period of needing care**														
< 2 years	463	96.3	18	3.7	5.8298			52	80.0	13	20.0	0.1893	0.6635	
2 years≦	530	92.8	41	7.2				109	77.3	32	22.7			
**Caregiver’s sex**														
Female	384	91.4	36	8.6	2.1439	0.1431		127	77.3	38	22.7	0.4310	0.5115	
Male	182	94.8	10	5.2				28	82.4	6	17.7			
**Caregiver/applicant dyad**														
Wife/Husband (living with)	190	92.6	15	7.3	1.5985	0.2061		76	76.8	23	23.2	0.3554	0.5511	
Others	787	94.9	42	5.1				85	80.2	21	19.8			
**Clients living with family or not**														
Living alone	498	96.3	19	3.7	7.0986	0.0077**		18	81.8	4	18.2	0.1730	0.6775	
Living with family	497	92.6	40	7.5				145	78.0	41	22.0			
**Corporation type of care management agency**														
Medical	259	91.8	23	8.2	4.6333	0.0314*		57	76.0	18	24.0	0.2700	0.6033	
Non-medical	748	95.3	37	4.7				106	79.1	28	20.9			
**Home-help service use**														
Yes	319	92.7	25	7.3	2.5861	0.1078		57	78.1	16	21.9	0.0006	0.9813	
No	688	95.2	35	4.8				106	77.9	30	20.1			

* p < 0.05 ** p < 0.01.

† Fisher’s exact test, other using the chi-squared test.

1 Lower income level: not paying personal income tax.

2 Higher income level: paying personal income tax.

### Determinants of VNS use: results of logistic regression analysis

The results of the stepwise multiple logistic regression analysis are shown in Table 5. The independent variables that are statistically significant in bivariate analysis are those with p-values less than 0.2: sex, the conditions that caused the need for LTC, regular hospital visits before entry into the LTCI, period of needing care, clients living with family or not, corporation type of care management agency and home-help service use in the lower care-needs group; age, income level and regular hospital visits before entry into the LTCI in the higher care-needs group. Sex, age, and the dummy variables that were created from the three care-needs levels as control variables were included in the model. In addition, we excluded the caregiver’s sex in the model due to multiple missing data. Through logistic regression analysis, we identified six factors in the lower care-needs level group and three factors in the higher care-needs level group that are associated with VNS use. In the lower care-needs level group, the following factors were related to likelihood of VNS use: the higher care-needs level, the condition that caused the need for long term care was respiratory or heart disease, the creation of a care plan by a medical care management agency, the period of needing care was two years or more, client living with family, and home-help service use (Table 5). In the higher care-needs level group, the following factors were related to the likelihood of VNS use: higher care-needs level, higher income level, and regular hospital visits before entry to the LTCI (Table 6).

**Table 5:** Multiple adjusted odds ratios of stepwise logistic regression analyses on the use of VNS in lower care level (Care support or care levels 1–2)

	**OR**	**95% CI**
Sex (female = 1, male = 0)	1.10	0.57–2.02
Age (under75 = 1, 75 and over = 0)	1.12	0.54–2.56
Care-needs level		
Support-requested level	1.00*	
Care-needs level 1	1.53	0.70–3.74
Care-needs level 2	3.50	1.50–8.93
Conditions that caused the need for LTC (Respiratory or heart disease = 1, others = 0)	4.31	1.88–9.20
Corporation type of care management agency (medical = 1, non-medical = 0)	2.39	1.31–4.33
Period of needing care(< 2 years = 0, ≧2 years = 1)	2.01	1.14–3.78
Clients living with family (yes = 1, no = 0)	1.83	1.00–3.42
Home-help service use(yes = 1, no = 0)	2.12	1.17–3.83
Goodness-of-fit statistics^1^ (*χ*^2^, p)	8.20	0.41

1 The Hosmer and Lemeshow Goodness-of-Fit Test.

* Reference.

**Table 6:** Multiple adjusted odds ratios of stepwise logistic regression analyses on the use of VNS in higher care level (Care levels 3–5)

	**OR**	**95% CI**
Sex (female = 1, male = 0)	0.92	0.42–1.99
Age (under75 = 1, 75 and over = 0)	1.82	0.79–4.51
Care-needs level		
Care-needs level 3	1.00*	
Care-needs level 4	1.39	0.58–3.30
Care-needs level 5	3.63	1.56–8.66
Income level (higher income level = 1, lower income level = 0)	3.79	1.01–14.25
Regular hospital visits before entry into the LTCI (yes = 1, no = 0)	2.36	1.11–5.38
Goodness-of-fit statistics^1^ (*χ*^2^, p)	9.41	0.22

1 The Hosmer and Lemeshow Goodness-of-Fit Test.

* Reference.

According to the results from the Hosmer-Lemeshow goodness-of-fit test, the model designed in this study has revealed that the VNS predictability was 0.41 for lower care level group and 0.22 for higher care level group.

## Discussion

Only 8.3% of total community-based service users utilized VNS, compared to the 32.7% of these users who used home-help services. Our study found that the following individuals are less likely to use the VNS: those who used a non-medical care management agency, those who lived alone in the lower care-needs level group or those in lower income in the higher care-needs level group. These results support our hypothesis that the present LTCI systems reduce the accessibility of the VNS. In the following, we shall discuss three issues related to the LTCI system.

First, LTCI’s care management system may not have been working effectively to support the VNS. Our results show that a care plan established by a medical care management agency was related to the likelihood of the VNS use in the only lower care-needs level group, not in the higher care group. It might be difficult for the non-medical care management agencies which comprise 90% of all care management agencies [20], to grasp the actual need for VNS and to recommend VNS to clients, especially to clients from the lower care-needs level group. A previous study reported that care plans arranged by care managers from non-medical corporations tend to include only a combination of social services [7]. Our findings regarding unequal access to VNS may facilitate discussions in other countries that consider the introduction of public LTCI. For example, in 2008, Germany started a care adviser service as new care management program that is partly based on Japan’s experience. Our results might be helpful for continuous discussions of introducing a new care management system under the public LTCI.

Second, living with family or home-help service use was related to the VNS in only the lower care-needs level group. Our results suggest that if there are caregivers around (either family or home-help services), access to VNS is facilitated. In addition, the significant relation between the users of home-help services and those of VNS was significant only in cases of living alone (data is not shown).In case of clients living with family, the clients’ daily living needs might be fulfilled by family caregiver and they can use VNS without using home-help services. On the other hand, clients who live alone might first request for daily life support from home-help services. After fulfilling their daily needs by home-help services use, they could use VNS in addition to home-help services, if the latter affordable. The home helpers might also recommend the use of VNS in such cases of clients living alone. According to the 2010 government survey on living conditions, single-person households make up 26.1% of the total households that need care, a 10.4 point increase from the 2001 survey [21]. The number of single elderly who needs care is expected to increase in the future. The results show that elderly people who have care needs can no longer rely on their families, and for that reason, policy makers should consider supporting elderly people who live alone at home with health services needs through the LTCI system.

Third, our results indicated that the present LTCI system has the potential to limit the use of VNS for low-income elderly people who have higher care needs. The clients who have higher care needs are more likely to use more services. Our results suggest that low-income individuals who have high care needs might have been prevented from the use of the high-priced VNS due to the economic burden of 10% user copayment.

Lastly, whereas the type of disease does play a role in VNS use in the lower care levels, this is not the case for the higher care levels. It seems that a more general level of illness-severity (expressed by regular hospital visits before LTCI) is more pivotal for VNS use.

Although we found meaningful results that could lead to an improvement of the Japanese elderly care system in the future, there were some limitations in the current study. First, this cross-sectional study may be limited by the study location, that is, a Japanese rural area. Average utilization of community-based services in the studied rural area was 67.2%, which was not drastically different from the national average (71.2%, in 2000) [22]. However, the generalization of our findings needs to be confirmed by similar studies in other regions, especially in the urban areas. In addition, because of the cross-sectional nature of our study, we could not directly evaluate the individuals who cancelled their use of the VNS. A longitudinal follow-up and investigation of other regions and populations are suggested. Next, we also should discuss that an influence of the type of diseases was not clearly demonstrated in our study. This could be affected by the fact that the information about diseases was subjectively answered by the users or family, and only one disease was selected. We should analyze this point with more exact and detailed data in future. Lastly, there may be a slight concern about 10 years old datasets used for this current study. The Japanese public long-term care insurance system performed a significant amendment on 2006 to shift on more prevention. However, this reform was only for support levels and some provision of care-needs level made no drastic change during the past ten years. Therefore, we believe that there should not be a remarkable change on data between the present results and current status.

## Conclusions

The Japanese LTCI eligibility criteria for service use are based on physical and mental status only [2,10]. We found, however, that individuals within the lower care level group who used the services of a non-medical care management agency to create a service plan, those who live alone, and those who used a corporate type of care management agency were less likely to use VNS. In lower care levels, our results suggest that VNS use is limited due to the corporation type of care management agency related to the unique services prevision system in Japanese LTCI. In addition, if there are caregivers around (either family or home help), access to VNS is better facilitated compared to those who depend on care management agencies alone. That is, after daily duties have been fulfilled by family or home-help service use, the clients may use VNS, if VNS are affordable. In higher care levels, income level or no regular hospital visit before entry into the LTCI were main factor to limit the VNS use. Low-income individuals who have high care needs might have been prevented from using high-priced VNS due to the economic burden of the 10% user copayment. If Japan adheres to the basic stance of providing equal access to health services through public insurance, LTCI policy makers should reconsider the present provision of VSN.

## Abbreviations

LTCI: Long term care insurance; VNS home: Visit nursing services.

## Competing interests

The authors declare that they have no competing interests.

## Authors’ contributions

MK worked on the following: the structure of the study design, statistical analysis, analysis, interpreting the data, and drafting the manuscript. NT supervised the entire process; NT also participated in the following: the design of the study, statistical analysis, interpretation of the data, and helped to finalize the manuscript. MS carried out special advice to structure the data set. EY supervised the entire study and provided academic guidance throughout the study process. All authors read and approved the final manuscript.

## Pre-publication history

The pre-publication history for this paper can be accessed here:

http://www.biomedcentral.com/1471-2318/13/1/prepub

## References

[B1] Cabinet Office, Government of Japan Annual Report on the Aging Society 2010 in Japanese). http://www8.cao.go.jp/kourei/english/annualreport/2010/2010pdf_e.html

[B2] Ikegami N Public long-term care insurance in Japan J Am Med Assoc 1997 278 16 1310 1314 10.1001/jama.1997.03550160030017 9343449

[B3] Campbell JC Ikegami N Long-term care insurance comes to Japan Health Affairs (Millwood) 2000 19 3 26 39 10.1377/hlthaff.19.3.26 10812779

[B4] Ministry of Health, Labour and Welfare Survey of Long-term Care Benefit Expenditures, 2001–2010 (in Japanese). http://www.mhlw.go.jp/toukei/list/45-1b.html

[B5] Ministry of Health, Labour and Welfare National medical care expenditure 2009 (in Japanese). http://www.mhlw.go.jp/toukei/list/37-21.html

[B6] Ministry of Health, Labour and Welfare Comprehensive Survey of Living Conditions 2010 (in Japanese). http://www.mhlw.go.jp/toukei/saikin/hw/k-tyosa/k-tyosa10/

[B7] Japan Medical Association Research Institute Report No.56 2003 (in Japanese). http://www.jmari.med.or.jp/research/summ_hb.php?no=87

[B8] Murashima S Nagata S Magilvy JK Fukui S Kayama M Home care nursing in Japan: a challenge for providing good care at home Public Health Nurs 2002 19 2 94 103 10.1046/j.1525-1446.2002.19204.x 11860594

[B9] Nagata S Taniguchi A Naruse T Kuwahara Y Murashima S Actual situation and characteristics of clients judged to need home-visiting nurse services by certified care managers and comparison of users and non-users of such services Nippon Koshu Eisei Zasshi 2010 57 1084 1093 in Japanese 21348283

[B10] Tamiya N Yamaoka K Yano E Use of home health services covered by new public long-term care insurance in Japan: impact of the presence and kinship of family caregivers Int J Qual Health Care 2002 14 4 295 303 10.1093/intqhc/14.4.295 12201188

[B11] Kemper P The use of formal and informal home care by the disabled elderly Health Serv Res 1992 27 421 451 1399651 PMC1069888

[B12] Solomon DH Wagner DR Marenberg ME Acampora D Cooney LM Inouye SK Predictors of formal home health care use in elderly patients after hospitalization J American Geriatric Soc 1993 41 961 966 10.1111/j.1532-5415.1993.tb06762.x 8409182

[B13] Logan JR Spitze G Informal support and the use of formal services by older Americans J Gerontol 1994 49 S25 S34 10.1093/geronj/49.1.S25 8282986

[B14] Houde SC Predictor of elders’ and family caregivers’ use of formal home services Res Nurs Health 1998 21 533 543 10.1002/(SICI)1098-240X(199812)21:6<533::AID-NUR7>3.0.CO;2-I 9839798

[B15] Picone SC Wilson RM Medicare home health agency utilization, 1984–1994 Inquiry 1999 36 291 303 10570662

[B16] Andersen R Newman JF Societal and individual determinants of medical care utilization in the United States Milbank Mem Fund Q Health Soc 1973 51 1 95 124 10.2307/3349613 4198894

[B17] Kato G Tamiya N Kashiwagi M Sato M Takahashi H Relationship between home care service use and changes in the care needs level of Japanese elderly BMC Geriatr 2009 9 58 10.1186/1471-2318-9-58 20025749 PMC2808302

[B18] Hosmer DW Lemeshow S Applied Logistic Regression 1989 Wiley, NY

[B19] Statistical Analysis System. Software Release 8.1.3 2012 SAS Institute, Inc (in Japanese). http://www.sas.com/jp/

[B20] Ministry of Health, Labor and Welfare The Survey of Institutions and Establishments for Long-term Care 2003 (in Japanese). http://www.mhlw.go.jp/toukei/saikin/hw/kaigo/service03/index.html

[B21] Ministry of Health, Labour and Welfare Comprehensive Survey of Living Conditions 2010 (in Japanese). http://www.mhlw.go.jp/toukei/list/20-21.html

[B22] Ministry of Health, Labour and Welfare Survey of long term care benefit expenditures 2001 (in Japanese). http://www.mhlw.go.jp/toukei/saikin/hw/kaigo/kyufu/01/index.html

